# Serum Uric Acid Levels Are Associated with Polymorphism in the *SAA*1 Gene in Chinese Subjects

**DOI:** 10.1371/journal.pone.0040263

**Published:** 2012-06-29

**Authors:** Xiang Xie, Yi-Tong Ma, Yi-Ning Yang, Xiao-Mei Li, Zhen-Yan Fu, Ying-Ying Zheng, Xiang Ma, Bang-Dang Chen, Fen Liu, Ying Huang, Zi-Xiang Yu, You Chen

**Affiliations:** 1 Department of Cardiology, First Affiliated Hospital of Xinjiang Medical University, Urumqi, People's Republic of China; 2 Xinjiang Key Laboratory of Cardiovascular Disease Research, Urumqi, People's Republic of China; University of Texas Health Science Center San Antonio Texas, United States of America

## Abstract

**Objective:**

Serum uric acid (SUA) is a cardiovascular risk marker associated with inflammation. The serum amyloid A protein (SAA) is an inflammatory factor and is associated with cardiovascular disease (CVD). However, the relationship between genetic polymorphisms of SAA and SUA levels has not been studied. The objective of this study was to investigate the association between SUA levels and SAA genetic polymorphisms.

**Methods:**

All participants were selected from subjects participating in the Cardiovascular Risk Survey (CRS) study. The single nucleotide polymorphism (SNP) rs12218 of the *SAA1* gene was genotyped by using the polymerase chain reaction–restriction fragment length polymorphism (PCR-RFLP) method. The association of SUA levels with genotypes was assessed by using the general liner mode.

**Results:**

The SNP rs12218 was associated with SUA levels by analyses of a dominate model (*P* = 0.002) and additive model (*P* = 0.005), and the difference remained significant after adjustment of sex, age, obesity, ethnicity, HDL-C, alcohol intake, smoking, and creatinine (*P* = 0.006 and *P* = 0.023, respectively). The TT genotype was associated with an increased SUA concentration of 39.34 mmol/L (95% confidence interval [CI], 3.61–75.06, *P* = 0.031) compared with the CC genotype, and the TT genotype was associated with an increased SUA concentration of 2.48 mmol/L (95% CI, 6.86–38.10; *P* = 0.005) compared with the CT genotype.

**Conclusions:**

The rs12218 SNP in the *SAA*1 gene was associated with SUA levels in Chinese subjects, indicating that carriers of the T allele of rs12218 have a high risk of hyperuricemia.

## Introduction

Serum uric acid (SUA) is the final product of purine metabolism in humans and higher primates. It has a well-established role in gout and is associated with other forms of inflammation and immune regulation as well [1]. The results of recent studies suggest that hyperuricemia is associated with inflammation [2], [3]. A high level of SUA is associated with hypertension [4], [5], insulin resistance [6], [7], obesity [6], [8] and cardiovascular events [5], [9]. A potential mechanism by which uric acid could be associated with cardiovascular morbidity is via inflammation [10]. Experimental results show that SUA can stimulate the release of C-reactive protein (CRP) when entering vascular smooth muscle cells [11]–[12].

Serum amyloid A (SAA), one of the acute phase proteins, is an important inflammatory factor. The results of a previous study [13] show that in vivo concentrations of SAA can be dramatically increased (up to 1000-fold) in plasma during acute inflammatory conditions [14]. However, the relationship between SUA and SAA is unknown. In previous studies, we observed that the single nucleotide polymorphism (SNP) rs12218 in the *SAA1* gene was associated with increased carotid artery intima – media thickness (IMT) values [15] and decreased HDL-C levels [16] and Ankle-to-Brachial Index [17], which are considered to be risk factors for cardiovascular disease (CVD). SUA levels were also associated with the risk of CVD via inflammation [10]. And SAA has been reported to be a more sensitive marker of inflammation and CVD than C-reactive protein (CRP) [13], [14]. Therefore, we hypothesized that rs12218 of SAA1 is associated with SUA levels.

## Methods

This study was approved by the Ethics Committee of the First Affiliated Hospital of Xinjiang Medical University and was conducted according to the standards of the Declaration of Helsinki. Written, informed consent was obtained from the participants.

### Subjects

All the participants were selected from the Cardiovascular Risk Survey (CRS) study, which was described previously [15], [17]. Briefly, the CRS included 14 618 participants (5 757 Hans, 4 767 Uygurs, and 4 094 Kazakhs). Of these 14 618 subjects, 1078 participants who were free from CVD were initially screened for the present study. The exclusion criteria were as follows: systolic and diastolic blood pressure (BP) ≥140/90 mmHg; fasting plasma glucose >7.0 mmol/L; total cholesterol >7.8 mmol/L; triglycerides >2.0 mmol/L; or electrocardiography (ECG) abnormalities and carotid artery plaques. Of the 1078 screened, 107 were excluded, leaving us with 971 subjects for the present study. Among these 971 subjects, only 568 consented to providing blood samples for DNA analysis. The analysis presented in the present study was based on these 568 participants who met the eligibility criteria and for whom complete SAA1 genotype data were available.

**Table 1:** Demographic and Risk Profile of the Study Population.

Risk factor	No. (%) or Mean (SD)		
	Total cohort (n = 658)	Men (n = 450)	Women (n = 118)
Age (years)	57.24 (11.84)	57.18 (12.28)	57.46 (10.01)
Obesity	150 (26.40)	124 (27.56)	26 (22.03)
Never drink (%)	312(54.9)	237 (52.7)	75 (63.6)
Former drinker (%)	154(27.1)	114 (25.3)	40 (33.9)
Current drinker (%)	102(18.0)	99 (22.0)	3 (2.5)
Never smoking (%)	213 (37.5)	136 (30.2)	77 (65.3)
Former smoking (%)	153 (26.9)	114 (25.3)	39 (33.1)
Current smoking (%)	202 (35.6)	200 (44.4)	2 (1.7)
BUN (mmol/L)	5.16 (1.53)	5.27 (1.56)	4.76 (1.36)
Cr (mmol/L)	75.29 (19.96)	78.91 (20.03)	61.49 (12.21)
Uric acid (µmol/L)	321.32(92.68)	336.18 (93.63)	264.68 (62.46)
Glucose (mmol/L)	4.69 (0.82)	4.67 (0.85)	4.75 (0.68)
Triglyceride (mmol/L)	1.81 (1.39)	1.86 (1.43)	1.65 (1.23)
Total cholesterol (mmol/L)	4.58 (1.16)	4.58 (1.18)	4.61(1.10)
HDL –C (mmol/L)	1.40 (0.46)	1.39 (0.47)	1.45 (0.39)
LDL-C (mmol/L)	3.06(1.0)	3.06 (0.98)	3.09 (1.07)

Note: HDL, high-density lipoprotein; LDL, high-density lipoprotein;

**Table 2:** Distribution of rs12218 in Han, Uygur, and Kazak population.

Rs12218	Ethnicity			P value
	Han (n = 321)	Uygur (n = 115)	Kazak (n = 132)	
CC, n (%)	19 (5.92)	1 (0.87)	8 (6.06)	0.012
CT, n (%)	124 (38.63)	64 (55.65)	57 (43.18)	
TT, n (%)	178 (55.45)	50 (43.48)	67 (50.76)	

**Table 3:** Demographic and Risk Profile of the Study Population According to rs12218 Genotypes.

Risk factor	rs12218 genotypes [No. (%) or Mean (SD)]			p
	CC (n = 28)	CT (n = 245)	TT (n = 295)	
Age (years)	56.43(12.07)	58.47(11.77	56.3(11.82	0.100.
Obesity	8 (28.57)	67 (27.35)	75 (25.42)	0.850
Never drink (%)	12 (42.86)	147 (60.0)	153 (51.86)	0.004
Former drinker (%)	15 (53.57)	56 (22.86)	83 (28.13)	
Current drinker (%)	1 (3.57)	42 (17.14)	59 (20.0)	
Never smoking (%)	10 (35.71)	101 (41.22)	102 (34.58)	0.557
Former smoking (%)	8 (28.57)	59 (24.08)	86 (29.15)	
Current smoking (%)	10 (35.71)	85 (34.69)	107 (36.27)	
BUN (mmol/L)	5.02(1.44)	5.22(1.67)	5.13(1.40)	0.709
Cr (mmol/L)	70.94(23.3)	74.21(18.95)	76.6(20.40)	0.191
Uric acid (µmol/L)	293.62(92.89)	310.48(89.8)	332.96(93.68)	0.005
Glucose (mmol/L)	4.69(0.64)	4.72(0.83)	4.65(0.81)	0.675
Triglyceride (mmol/L)	1.63(0.85)	1.79(1.43)	1.85(1.39)	0.683
Total cholesterol (mmol/L)	4.47(1.17)	4.62(1.28)	4.56(1.04)	0.710
HDL –C (mmol/L)	1.26(0.40)	1.41(0.49)	1.47(0.42)	0.042
LDL-C (mmol/L)	3.24(1.23)	3.05(1.03)	3.05(0.94)	0.619

Note: HDL, high-density lipoprotein; LDL, high-density lipoprotein;

### Biological and lifestyle measurements

Height and body weight were measured as described previously [18]. Sitting blood pressure was measured three times during 10 min, and the median value was used for statistical analysis. Smoking and drinking status was self-reported via the study questionnaire as described previously [19], [20]. Body mass index (BMI) was calculated by dividing the weight in kilograms by the height in meters squared. Obesity was defined as BMI ≥25 kg/m2 which was based on the WHO Asia-Pacific Area criterion for obesity described previously [18]. We measured the serum concentration of uric acid, total cholesterol, triglyceride, blood urea nitrogen (BUN), creatinine (Cr), low density lipoprotein (LDL), high density lipoprotein (HDL), and fasting glucose using chemical analysis equipment (Dimension AR/AVL Clinical Chemistry System, Newark, NJ) in the Clinical Laboratory Department of the First Affiliated Hospital of Xinjiang Medical University as described previously [15]–[17], [19], [20].

**Table 4:** SUA levels and SAA1 genotypes.

SNP	Unadjusted model		Adjusted model§	
rs12218 Genotypes	SUA (mmol/L) Mean (SD)	*P* value	SUA (mmol/L) Mean (SE)	*P* value
Additive model				
CC (n = 28)	293.62 (92.89)	0.005	309.72 (15.25)	0.023
CT (n = 245)	310.48 (89.80)		312.10 (5.15)	
TT (n = 295)	332.96 (93.68)		330.49 (4.68)	
Dominant model				
CC+CT (n = 273)	308.75 (90.09)	0.002	311.86 (4.87)	0.006
TT (n = 295)	332.96 (93.68)		330.49 (4.68)	
Recessive model				
CC (n = 28)	293.62 (92.89)	0.105	309.88 (15.33)	0.436
CT+TT (n = 540)	322.76 (92.53)		322.16 (3.47)	

§ Analysis of covariance adjusted for sex, age, Cr, obesity, ethnicity, HDL-C, smoking and drinking.

**Table 5:** Interaction of rs12218 and other confounders on SUA.

Source	Squares	df	Mean Square	F	P
Corrected Model	1.801×10^−6^	19	94787.509	17.145	<0.001
Rs12218	14194.811	2	7097.406	3.155	0.043
Age	27856.254	1	27856.254	5.039	0.025
BUN	27943.440	1	27943.440	5.054	0.025
Glucose	7013.364	1	7013.364	1.269	0.261
Cr	403270.745	1	403270.745	72.944	<0.001
Triglyceride	201494.695	1	201494.695	36.446	<0.001
Total cholesterol	1215.210	1	1215.210	0.220	0.639
HDL-C	4810.028	1	4810.028	0.870	0.351
LDL-C	64303.867	1	64303.867	11.631	0.001
Sex	91370.100	1	91370.100	16.527	<0.001
Drinking	1869.215	1	1869.215	0.338	0.561
Obesity	42965.305	1	42965.305	7.772	0.005
Rs12218 * sex	11572.711	2	5786.355	1.047	0.352
Rs12218 * drinking	8397.275	2	4198.638	0.759	0.468
Rs12218* HDL-C	693.427	2	346.713	0.063	0.939

R Squared = 0.374 (Adjusted R Squared = 0.352).

### SAA1 SNP genotyping

As described in the previous study [17], there are 115 SNPs for the human *SAA 1* gene listed in the National Center for Biotechnology Information SNP database (http://www.ncbi.nlm.nih.gov/SNP). Using the Haploview 4.2 software and the HapMap phrase II database, we obtained four tagging SNPs (rs12218, rs4638289, rs7131332 and rs11603089) for Chinese Hans by using minor allele frequency (MAF) ≥0.05 and linkage disequilibrium patterns, with r ^2^≥0.8 as a cutoff. Because we found that only rs12218 was associated with CVD risks in the previous studies [15]–[17], we selected rs12218 for the present study. We genotyped rs12218 according to the protocol described previously [17]. To ensure the results were verified, 10% of the genotyped samples were duplicated, and at least one positive and one negative control per 96-well DNA plate were used in our assays. The accuracy of the genotyping was determined by assessing the genotype concordance between duplicate samples. We obtained a 100% concordance between the genotyped duplicate samples for the SNP. The genotyping success rate was 100%.

### Statistical analysis

All analyses were performed by using SPSS version 17.0 (SPSS, Chicago, IL, USA). Hardy-Weinberg equilibrium was assessed by chi-square analysis. Serum UA levels were normally distributed; therefore, the original values were used for analysis. General linear model (GLM) analysis was performed to test for associations between SNP genotypes and serum UA after adjusting for confounding variables. Two-tailed *P*-values were considered to be significant at the 0.05 level.

## Results

### Characteristics of study participants

The study cohort consisted of 568 subjects (450 men, 118 women). The clinical and metabolic characteristics of the study population are shown in Table 1. Table 2 shows the distributions of rs12218 in the Han, Uygur, and Kazak populations. Genotype distributions between each ethnicity were significantly different (*P* = 0.012). Table 3 shows the characteristics of study participants according to their rs12218 genotypes. Each genotype was significantly different in terms of SUA, HDL-C, and drinking behavior (*P*<0.05 for all comparisons).

### SAA1 genotype and SUA

The genotype frequencies for the rs12218 were in Hardy-Weinberg equilibrium (P>0.05). Table 4 shows that rs12218 was associated with SUA levels by analyses of a dominate model (*P* = 0.002) and an additive model (*P* = 0.005), and the difference remained significant after adjustment for sex, age, ethnicity, HDL-C, obesity, alcohol intake, smoking, and creatinine levels (P = 0.006, P = 0.023, respectively). The TT genotype was associated with an increased SUA concentration of 39.34 mmol/L (95% confidence interval [CI], 3.61–75.06, *P* = 0.031) compared with the CC genotype, and the TT genotype was associated with an increased SUA concentration of 2.48 mmol/L (95% CI, 6.86–38.10; *P* = 0.005) compared with the CT genotype. Taking into account the possible interactions between rs12218 and other variables, we analyzed all these variables using a general linear model and found no interactions between rs12218 and sex, alcohol intake, or HDL-C level (Table 5).

## Discussion

In the present study, we observed that variation in the *SAA*1 gene is associated with SUA in Chinese subjects. Individuals with the T allele of rs12218 had significantly higher SUA levels than did C allele carriers. To the best of our knowledge, this is the first study to investigate the common allelic variant in the *SAA*1 gene and its association with SUA levels.

Hyperuricemia has been implicated in multiple physiologic outcomes, including coronary artery disease, hypertension [4], [5], and obesity [6], [8]. Hyperuricemia is suspected to influence the development of CVD via its role in inflammation [10]. SAA is in a family of proteins that forms a major component of the acute-phase inflammatory response [21]. It is synthesized in the liver in response to inflammation and infection [22] and is considered to be a sensitive marker of an acute inflammatory state. Therefore, SUA and SAA may be associated. However, up to date, the relationship between SAA and SUA remains unclear.

Accumulated evidence generated from genome-wide association studies (GWAS) has linked uric acid to specific genomic loci, such as SLC2A9, PDZK1, GCKR, SLC16A9, SLC22A11, SLC22A12, and ABCG2, in individuals of European and Asian descent [23]–[26]. Although the foundation for human studies examining putative causative genes that may be involved in hyperuricemia is based on a GWAS method, the candidate-gene approach is an important way to explore the association of hyperuricemia with genetic polymorphisms. This approach involves selecting a functionally relevant gene to study and subsequently investigating its association with SUA levels. The gene for *SAA1* is a candidate hyperuricemia gene because it encodes the important inflammation factor SAA. This factor was identified in the early 1970s as the plasma protein responsible for forming tissue deposits called amyloid (AA-type), which are seen clinically in diseases with underlying persistent acute inflammation [27], [28].

In previous studies, we found that the rs12218 SNP in the *SAA1* gene was associated with IMT [15], HDL level [16], and ABI [17], which is involved in CVD. In the present study, we found that the rs12218 polymorphism is associated with SUA concentration, which is also related to CVD. Compared with C-allele carriers, individuals with the T allele of rs12218 had significantly higher SUA levels. This association remains significant after adjustment for sex, age, obesity, ethnicity, HDL-C, alcohol intake, smoking status, and creatinine levels.

The mechanisms which may link *SAA1* genetic polymorphisms to SUA levels are largely unknown. The results of previous studies show a close association between elevated SUA and several inflammatory markers such as white blood cells, TNF-α, and CRP [29]. Also, uric acid directly stimulates the production of inflammatory mediators such as CRP in vascular cells [30]. However, the relationship between SAA and SUA remains unclear. In the present study, we observed that the *SAA1* genetic polymorphism was associated with elevated SUA after adjusting for other confounders, indicating that the *SAA1* gene polymorphism may be independently related to SUA level. However, the relationship between rs12218 and SUA may simply reflect their association with other inflammatory factors. Therefore, functional studies of rs12218 should be performed in the future.

In addition, rs12218 is a synonymous mutation and does not result in amino acid changes in the protein. However, rs12218 acting as a tagging SNP of SAA1 gene may be in total linkage disequilibrium with a nonsynonymous mutation, which we did not examine. Because both SAA and SUA are related to inflammation, the association of *SAA1* polymorphism with SUA may be linked to SAA levels. In 1999, Yama et al. [31] reported that the SAA1 allele influences the plasma concentration of SAA. In the Japanese population, subjects with the SAA1.5 allele have a higher plasma concentration of SAA than those lacking this allele. In the present study, we did not examine the differences in SAA levels between each genotype, which was a limitation of our analysis. We think that this SNP may be associated with rs12218 although we could not identify the rs number of the SAA1.5 polymorphism described by Yama et al [29]. Dietary intake of certain macronutrients and food items may also significantly affect SUA concentration. However, because of the absence of these data in our database, we did not include these factors in our multivariable analysis, which is also a limitation of our study. We also did not perform experiments related to *SAA1* gene function. In addition, in the present study, we found that there was significantly difference in distributions of genotypes in Han, Uygur, and Kazak population. However, due to the very small sample size in Uygur and in Kazak population, we did not analyze the relationship between rs12218 and SUA in each ethnicity. Alternatively, we pooled these three ethnicities together to analyze the data and adjusted the effect of ethnicity by using multivariable analysis. This may be another limitation in our research.

In conclusion, a polymorphism of *SAA*1 gene was associated with the SUA level in Chinese subjects; our observation requires replication in a different population.
